# Editorial: Novel paradigms in cardiothoracic and abdominal disorders in veterinary practice

**DOI:** 10.3389/fvets.2024.1369276

**Published:** 2024-04-08

**Authors:** Ahmed S. Mandour, Ahmed Farag, Tomohiko Yoshida, Ryou Tanaka

**Affiliations:** ^1^Department of Animal Medicine (Internal Medicine), Faculty of Veterinary Medicine, Suez Canal University, Ismailia, Egypt; ^2^Veterinary Surgery, Tokyo University of Agriculture and Technology, Tokyo, Japan; ^3^Department of Surgery, Anesthesiology, and Radiology, Faculty of Veterinary Medicine, Zagazig University, Zagazig, Egypt; ^4^Department of Veterinary Surgery, Division of Veterinary Research, Obihiro University of Agriculture and Veterinary Medicine, Obihiro, Hokkaido, Japan

**Keywords:** cardiothoracic, ultrasound, echocardiography, biomarkers, diagnosis

Cardiothoracic and abdominal disorders constitute a large proportion of animal diseases and are the leading causes of considerable deaths in the field of veterinary medicine. There are continuous research trials to find out simple diagnostic methods to monitor internal organ functions effectively and easily with time-saving procedures, high accuracy, and minimum invasiveness. Moreover, recent treatment strategies including new therapeutic agents and/or surgical techniques as well as diagnostic biomarkers are currently highly developed. Recent research studies are focusing on new diagnostic imaging techniques such as novel ultrasonographic approaches, echocardiography-derived techniques, blood flow dynamics, etc., in addition to new surgical procedures and laboratory biomarkers. These trendy methods showed enormous potential to improve the outcome of cardiothoracic and abdominal disorders in animal models and clinical practice. In this Research Topic, nine articles were published covering the recent approaches in the aforementioned objectives which may serve as a guidance to researchers and veterinarians in the field.

The exploration of new treatments for heart failure involves evaluating potential therapeutic approaches in suitable animal models designed to mimic heart failure conditions. Over the recent decades, murine models of cardiovascular diseases have provided efficient strategies for preventing and managing cardiac dysfunctions. Establishing these models begins with precise surgical techniques and well-designed anesthetic protocols. Nevertheless, each protocol may exhibit limitations that can impact the results of the study. In this Research Topic, Farag, Mandour, Hendawy et al. wrote a systematic review covering the heart failure in murine models, considering the most common and recent surgical models of heart failure and the anesthetic protocols. Moreover, they listed the surgical procedures of each model, the proper anesthesia, and the limitations in each single model, which can guide the researcher during selection of the model.

In veterinary cardiology, novel anesthetic protocols that may save time, money, or overcome restriction on medicinal agents in certain countries are crucial. Numerous validated anesthetic agents used to create myocardial infarction (MI) model are currently controversial with certain restrictions due to ethical concerns. The combination of medetomidine, midazolam, and butorphanol (MMB) is frequently employed in various surgical operations in animals. However, there hasn't been an exploration of the use of the MMB combination to create the MI in rats. This is challenging due to the pronounced respiratory depression and prolonged recovery observed post-surgery, leading to substantial mortality rates. Farag, Mandour, Hamabe et al. established a new protocol of anesthesia to create MI in rat models using a combination of MMB (0.3/5.0/5.0 mg/kg) and atipamezole (1.0 mg/kg SC). Conclusively, subcutaneous administration of atipamezole effectively mitigates the cardiopulmonary side effects of the MMB mixture, facilitating rapid recovery and consequently enhancing the survival rate during the establishment of the MI model in rats.

Increasing evidence indicates that dental disorders contribute to the onset of cardiovascular disease. Several epidemiological studies have proposed a potential connection between periodontitis and cardiovascular diseases. In the current paper Research Topic, Elhaieg et al. used various comprehensive methods including conventional echocardiography, intraventricular pressure gradient analysis, Speckle Tracking Echocardiography, and invasive hemodynamic analysis to evaluate the heart function in rat model with periodontitis. This study suggests that periodontitis may compromise systolic function and myocardial relaxation.

Elevated pulmonary artery pressure is known as pulmonary hypertension (PH). Canine PH is commonly occurring secondary to myxomatous mitral valve disease (MMVD). Certain echocardiographic calculation as well as laboratory markers have been investigated in canine PH, however, they are still not accurate. Certain blood indices have previously been found to be indicators for prediction and prognosis of PH in human. Tangmahakul et al. examined the applicability of the blood indices in canine patients affected by MMVD with and without PH. The results confirmed a reduction in MCH and MCHC in dog patient suffering from MMVD, precapillary PH, and postcapillary PH, while PDW are associated with MMVD severity but not with the presence of PH.

In recent years, there has been a notable rise in the occurrence of hypertrophic cardiomyopathy (HCM) in feline patients within clinical practice, primarily attributed to advancements in diagnostic methods and equipment capabilities. Saito et al. evaluated myocardial function in cats affected by HCM with and without outflow obstruction (HOCM) using 2D speckle-tracking echocardiography. Their findings indicated that all HCM-affected cats exhibited a notable decrease in left ventricular (LV) longitudinal strain across the endocardial, epicardial, and overall layers, as well as in LV circumferential strain within the epicardium, in comparison to healthy cats. Cats with HOCM showed a significant reduction in both the endocardial and overall layers of LV circumferential strain when compared to healthy cats. Consequently, the diminished LV endocardial strain had a cascading effect on the values of LV strain across the entire myocardial layer, leading to the conclusion that LV myocardial function may be more compromised in HCM-affected cats with concurrent outflow obstruction.

Chronic idiopathic intestinal inflammation is a growing global health concern affecting both companion animals, especially dogs, and human patients leading to significant fluid and electrolyte losses. Interestingly, the differences observed in the handling of intestinal electrolytes in human and canine patients imply the existence of species-specific regulatory or counterregulatory mechanisms. In the context of preserving fluid and electrolyte balance, the renin-angiotensin-aldosterone system (RAAS) assumes a pivotal role. It is well-known that RAAS plays a systemic role in regulating blood pressure and cardiovascular pathology, however, it has unveiledcomplex roles in the realm of inflammatory processes. In the perspective article authored by Heilmann et al., they offered an overview of our current understanding of electrolyte transport in the context of human IBD and canine chronic inflammatory enteropathy. Additionally, they explore the role of RAAS in these conditions and propose innovative therapeutic strategies.

In the same line, Dengler et al. conducted a study to explore the gene expression of intestinal electrolyte transporters that may be implicated in either mitigating or exacerbating electrolyte losses in dogs with chronic idiopathic enteropathy. They also investigated the potential activation of the RAAS system in these dogs and explored the potential associations between the expression of intestinal electrolyte transporters and established RAAS components. Serum RAAS fingerprint analysis, mRNA levels of intestinal electrolyte transporters, and local RAAS pathway components in tissue biopsies were analyzed. The results indicated increased levels of components from both the traditional and alternative RAAS pathways in dogs with chronic idiopathic enteropathy. The study illustrated an upregulation of both traditional and alternative components of RAAS in the serum of dogs with chronic idiopathic enteropathy.

Park et al. documented an intriguing case involving 9-month-old female Pomeranian dog suffering from cyst-like lesions caused by generalized lymphatic anomaly (GLA) which involve several abdominal organs. The histopathological analysis and immunohistochemistry (IHC) confirmed that the cells lining these cyst-like lesions strongly expressed lymphatic vessel endothelial hyaluronan receptor 1. Notably, GLA should be considered in young dog when presenting with multiple cysts in various abdominal organs.

Congenital lobar emphysema (CLE) is an infrequent lower respiratory tract disorder that predominantly manifests in young dogs and cats. Edwards et al. detailed the evaluation and treatment of an 11-week-old, sexually intact female Catahoula Leopard dog presented with exercise intolerance and respiratory distress. There was hyperinflated right middle lung field, resulting in the compression of surrounding lung lobes. Following lung lobectomy, histopathology revealed the presence of bronchial cartilage hypoplasia, marked emphysema, and pleural fibrosis.

The investigations in this Research Topic have demonstrated the state of knowledge regarding the diagnosis and management of cardiothoracic disorders in cats and dogs. We anticipate that these articles will stimulate and motivate additional research into the development of cutting-edge diagnostic imaging novel echocardiography methods and the biofluid analysis of cardiac biomarkers.

## Author contributions

AM: Writing—review & editing, Writing—original draft, Validation, Supervision, Methodology, Investigation, Data curation, Conceptualization. AF: Writing—review & editing. TY: Writing—review & editing. RT: Writing—review & editing.

